# Novel cementitious materials with mechanoluminescence for the application of visible stress monitoring and recording

**DOI:** 10.1038/s41598-023-34500-5

**Published:** 2023-05-24

**Authors:** Bing Zhang, Shiqi Liu, Zichen Zhou, Ming Zeng, Jianfeng Zhang, Dong Tu

**Affiliations:** 1 Wuhan Research Institute of Metallurgical Construction, MCC, Wuhan, 430081 China; 2 Faculty of Materials Science and Chemistry, China University of Geosciences, 388 Lumo Road, Wuhan, 430074 China; 3 Wuhan University Shenzhen Research Institute, Shenzhen, 518057, China

**Keywords:** Engineering, Chemical engineering, Civil engineering

## Abstract

The development of real-time and accurate visual stress detection is crucial for the field of building engineering. Herein, a new strategy is explored for the development of novel cementitious materials by hierarchical aggregation smart luminescent material and resin-based material. The cementitious material with such layered structure is inherently capable of visualization of stress monitoring and recording by converting the stress to visible light. The specimen fabricated by the novel cementitious material could repetitively emit green visible light under excitation of a mechanical pulse for 10 cycles, suggesting that the cementitious material shows highly reproducible performance. Moreover, the numerical simulations and analysis for the models of stress indicate that the luminescent time is synchronous with the stress and the emission intensity is proportional to the value of stress. To the best of our knowledge, this is the first study that the cementitious material realizes visible stress monitoring and recording, which supplies new insights for exploring modern multi-functional building materials.

## Introduction

Cementitious materials play a very important role in the development of human civilization, especially for the modern construction field^1–5^. However, the structures of building are becoming more and more complex with the improvement of structural design and material processing capacity, which lead to the difficulty in detection of stress distribution on the structure. In addition, the distribution of stress will be changed due to the complex service environments such as alternating load, corrosion fatigue or cracks, which may result in fracture of structure. Currently, the stress detection methods mainly include electrical measurement method and grating method, which converts the strain signal into electrical signal or optical signal^6–12^. However, the current methods can only collect one point data at a time through specific instrument, it is difficult to quickly and accurately observe the stress distribution of the entire structure by the human eyes. Therefore, it is important to explore novel method to realize visual monitoring and recording of stress.

At present, it is a prevailing method to explore novel intelligence cementitious materials by adding smart materials^13–17^. Wherein, luminescent cementitious materials have been obtained by combining phosphor with cementitious material^18–20^. However, the current research only realizes visible light emission, and there is no in-depth application of visible light. It is well known that visible light is a very simple signal to be observed by human eyes, so if the cementitious material can convert the stress into visible light, the visual detection of stress can be realized. Mechanoluminescence (ML) material is a kind of emerging smart material, which can convert the external mechanical energy into light emission without the assistance of electron or photon excitation^21–26^. In recent years, researchers have developed many novel ML materials, such as ZnS/CaZnOS:Mn^21^, CaLaAl_3_O_7_:Tb^3+^^25^ and SrMgAl_10_O_17_:Ce^3+^^27^, etc. Our previous research found that the emission color of solid solution compounds (Ca_1−*x*_Sr_*x*_)_8_Mg_3_Al_2_Si_7_O_28_:Eu^2+^ could be tuned from green to blue under the elastic stress, which realized stress-induced ML spectral migration, and supplied new insights into the stress detection^28^. This kind of luminescent mode enables the ML material to be applied in the field of stress distribution. However, most ML materials are applied in the engineering by spraying film, the quality of spraying film gradually deteriorates as time goes on, which reduces the accuracy of the detection results. Moreover, the environment is complex in the cementitious material, such as high internal humidity and strong alkalinity, which can significantly affect the luminescent property of phosphor when the phosphor is doped into the interior of cementitious material. Thus, it is very important to explore appropriate method to introduce suitable ML material into the interior of cementitious material, so that the cementitious material can display multifunction and naturally convert the stress into visible light, which is conducive to convenient, rapid and accurate detection of stress in building structure.

In this study, resin is selected as the cementitious material (resin-based cementitious material) to aggregate ML material for preparation of novel resin-based cementitious material with visible stress monitoring and recording. The mechanical properties of resin-based cementitious materials were optimized by the doping of fibers due to its brittleness. The effects of different types of fibers and amounts were firstly discussed, and then the smart ML phosphor was added into the optimized cementitious material. The relationship between the distribution of stress and the visible light was investigated in detail. Moreover, the synthesized cementitious material also exhibited afterglow characteristic, the luminescent mechanisms of afterglow and mechanoluminescence were studied. The synthesized novel cementitious materials realized the direct observation of stress distribution through the visible light after molding, which presented high application values in building field.

## Results and discussion

**Figure 1:**
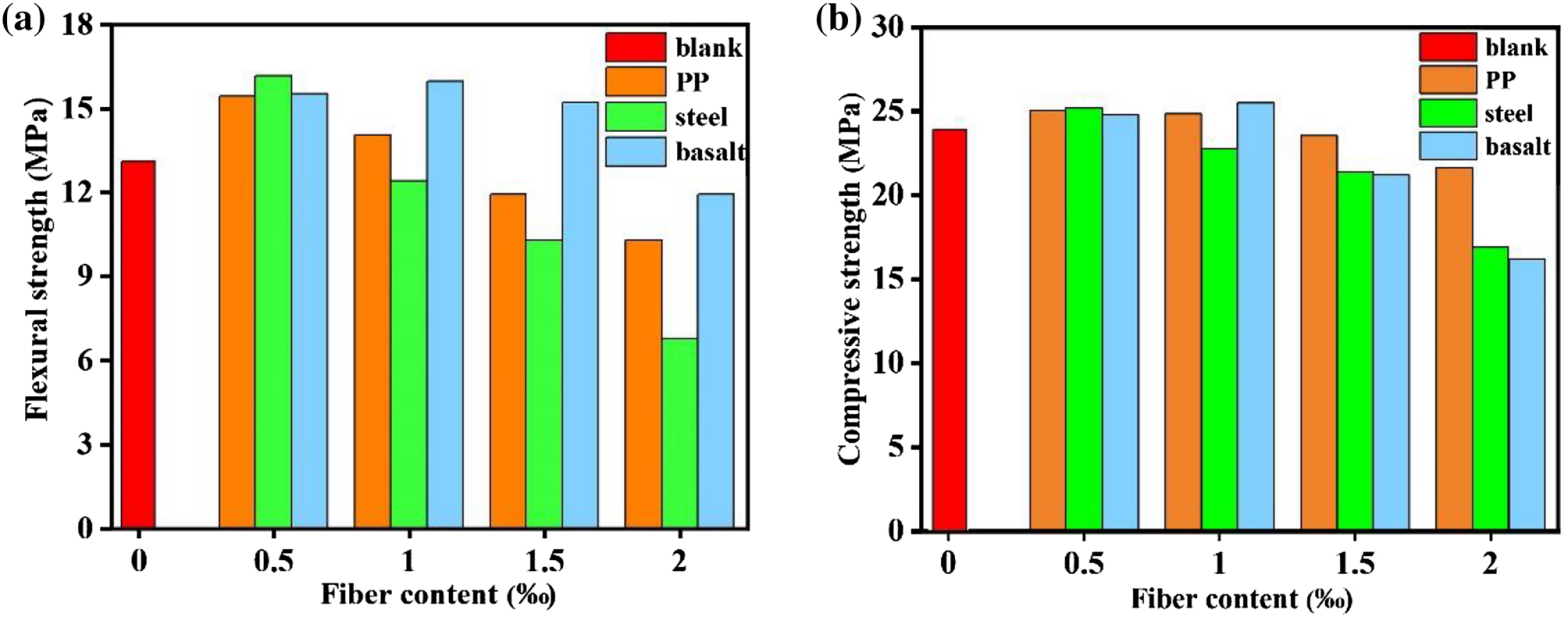
The flexural(**a**) and compressive(**b**) strengths of single fiber doped specimens.

The mechanical property of specimen is optimized by different kind of fibers. The mechanical strengths of resin specimen after PP, steel and basalt fiber reinforced were examined and are displayed in Fig. 1. Compared with the blank specimen, the flexural strength can be improved by 1.17, 1.23 and 1.18 times by the doping of 0.5 vol‰ PP, 0.5 vol‰ steel or 0.5 vol‰ basalt fibers, respectively (Fig. 1a). Similar to the flexural strength, the compressive strength is also increased after fiber reinforced. As shown in Fig. 1b, the compressive strength is enhanced by 1.05, 1.06 and 1.04 times for 0.5 vol‰ PP, 0.5 vol‰ steel or 0.5 vol‰ basalt fiber, respectively. It could be found that the mechanical strength of specimen can be improved after fiber reinforced, but the reinforcement degree is different for different kinds of fiber. Moreover, the flexural and compressive strengths of specimen decrease when the contents of PP and steel fiber exceeds 0.5 vol‰, which is different from that of basalt. This phenomenon may be due to the variable enhancement mechanism for different types of fiber^30–32^. Normally, the failure process of specimen under compressive relates to micro crack generation, expansion, macro crack formation and penetration. For the generation of initial micro crack, PP and basalt fibers play a bridging role to prevent the development of micro crack. When the micro crack continues to expand and form macro crack, the steel fiber begins to restrict the development of crack to avoiding the rapid collapse and failure of specimen^33–36^. Based on the different reinforced mechanisms, the doping of hybrid fiber is an efficient method to improve the mechanical strength of specimen.

**Figure 2:**
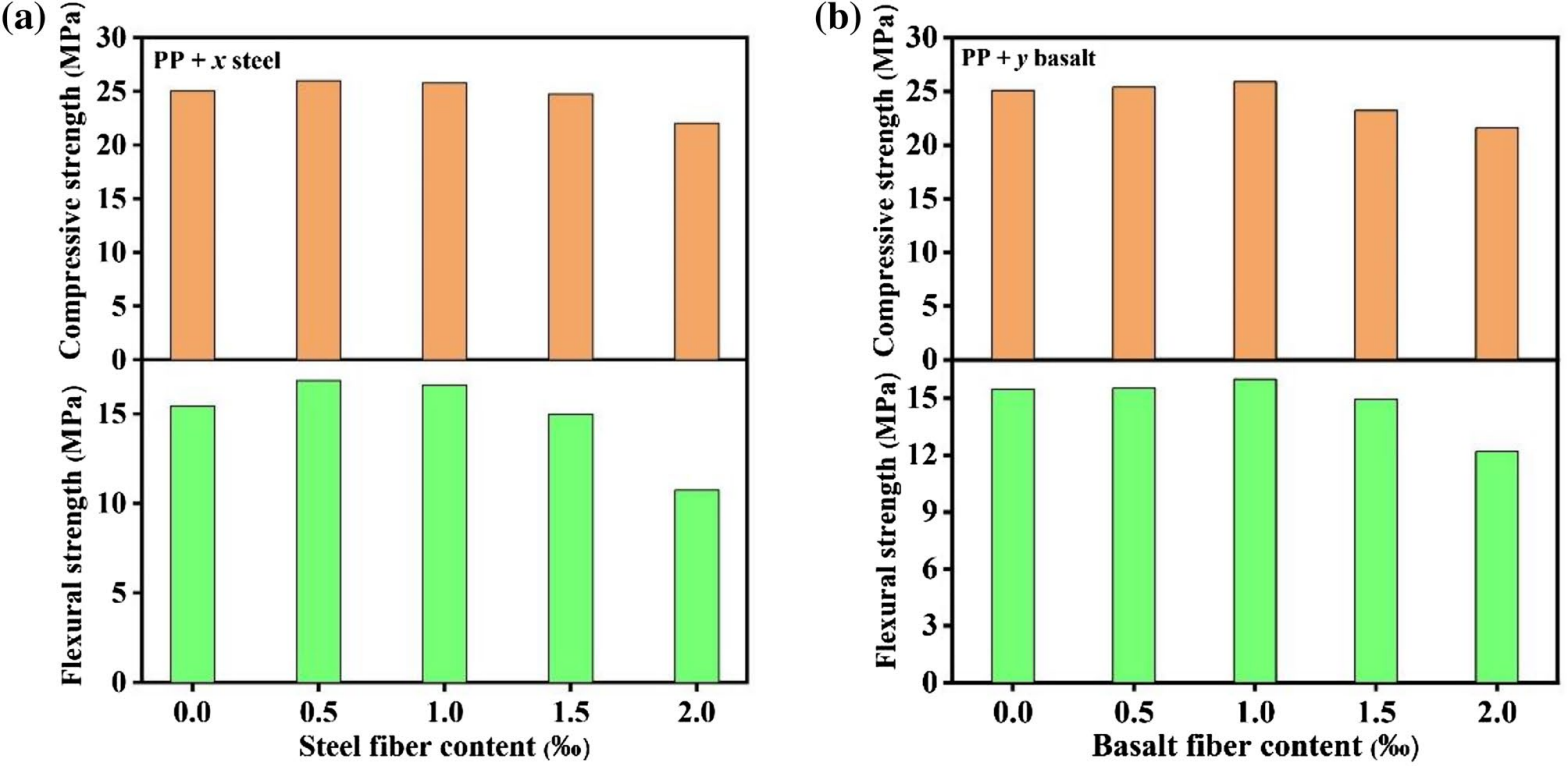
The flexural (**a**) and compressive(**b**) strengths of PP + *x*steel/*y*basalt hybrid fiber doped specimens.

Figure 2 presents the mechanical behaviors of specimen by doping of PP + *x* steel and PP + *y* basalt hybrid fiber. As shown in Fig. 2a, the flexural and compressive strengths of specimen doped with 0.5 vol‰ PP + 0.5 vol‰ steel hybrid fiber could be increased by 1.09 and 1.04 times compared with the PP single doped, indicating that the hybrid fiber is beneficial for the improvement of flexural and compressive strengths, which is assigned to prevention of the micro and macro cracks with the help of PP + steel hybrid fiber. However, it should be noticed that the doping content should be controlled within a reasonable range. As presented in Fig. 2a, the flexural and compressive strengths reduce to 0.70 times and 0.88 times of the initial strength when the *x* = 2.0 vol‰, which may be because the excessive contents increase the viscosity of the mixture, resulting in the uneven composition during molding and then affects the strength. Similarly, the PP + *y* basalt hybrid fiber can also improve the flexural and compressive strength of specimen, as shown in Fig. 2b, which could increase by up to 1.03 and 1.04 times compared with the initial sample.

**Figure 3:**
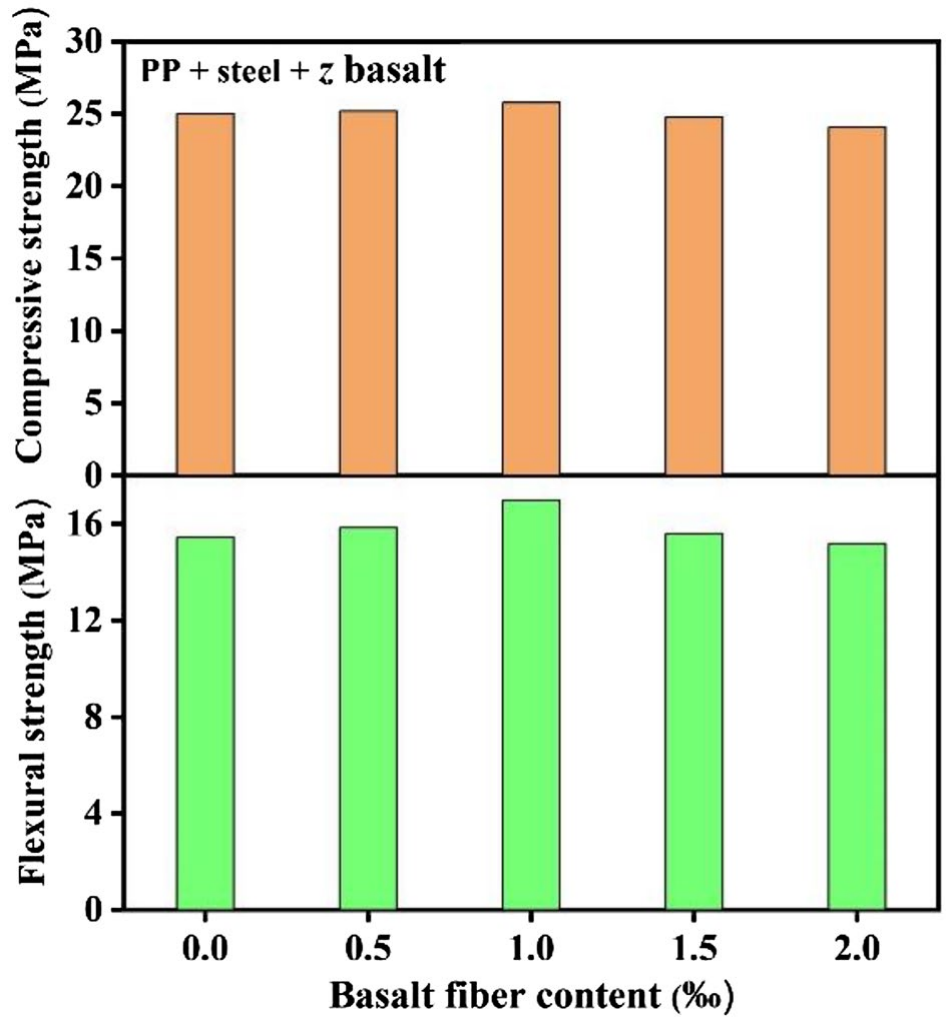
The flexural and compressive strengths of PP + steel + *z*basalt hybrid fiber doped specimens.

Based on the above results, it could be confirmed that the doping of hybrid fiber could improve the mechanical behavior through different dimensions. In order to optimize the mechanical property constantly, three kinds of fiber are blended and their effects on the strength are presented in Fig. 3. Both of the flexural and compressive strengths of specimen could be furtherly improved when the 1.0 vol‰ basalt is added to the 0.5 vol‰ PP + 1.0 vol‰ steel system. Compared with the initial sample, the flexural and compressive strengths increase by up to 1.10 and 1.02 times, respectively, and the improvement of flexural strength is better than that of compressive strength. The results suggest that the mechanical property of rubber modified resin specimen could be significantly improved by the 0.5 vol‰ PP + 1.0 vol‰ steel + 1.0 vol‰ basalt hybrid fiber. Based on the remarkable result, the ML material is added to the hybrid fiber optimized specimen to realize visible stress monitoring and recording. The flexural and compressive strengths of the specimen after phosphor doping are 15.98 Mpa and 24.75 Mpa respectively, which are close to that of initial specimen, indicating that the doping of phosphor displays little impact on the strength.

**Figure 4:**
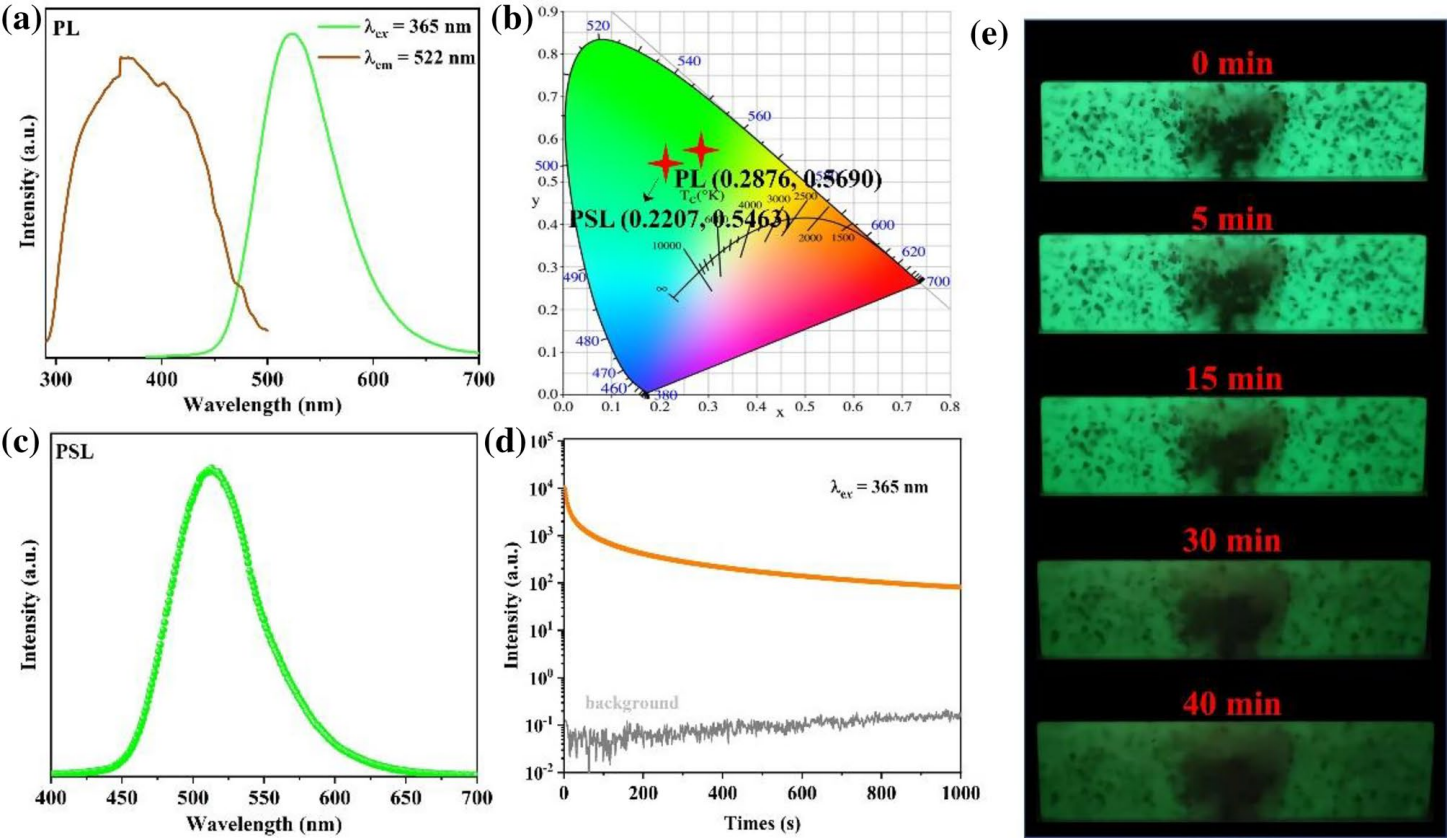
(**a**) PLE and PL spectra of specimen. (**b**) The CIE coordinates of PL and PSL. (**c**) The PSL spectrum of specimen. (**d**) The persistent luminescence intensities as a function of time. (**e**) Photos of the persistent luminescence by changing the delay time.

Next, the performance of specimen in different kinds of luminescent modes is tested. The photoluminescence (PL) and persistent luminescence (PSL) spectra of specimen were firstly examined and are presented in Fig. 4. Figure 4a shows the PL spectrum, which displays a broad green emission peak, located at 522 nm, The green emission responds to the transition of 5d-4f. for Eu^2+^^37,38^, and the CIE coordinate is (0.2876, 0.5690) (Fig. 4b). After analyzing the basic PL property, the long afterglow properties of specimen are tested. All the texts are examined in a dark room after irradiation by 365 nm light source for 10 min. Figure 4c presents the PSL spectrum, which comprises a broad green emission peak, centered at 513 nm. This phenomenon indicates that the PSL emission is also originated from Eu^2+^ emission, and the CIE coordinate locates at (0.2207, 0.5463) (Fig. 4b). The persistent decay time study reveals a long excited-state lifetime for the specimen (Fig. 4d). The decay curve decreases quickly in initial decay (200 s) before stabilizing to a slow decay even until 1000 s, where the emission intensity is still much higher than the background signal. Figure 4e shows the decay of emission colors for the specimen. When the UV light turns off, the specimen still displays green emission, which even can be observed after 40 min. The above results indicate that the synthesized specimen processes PL and PSL properties.

**Figure 5:**
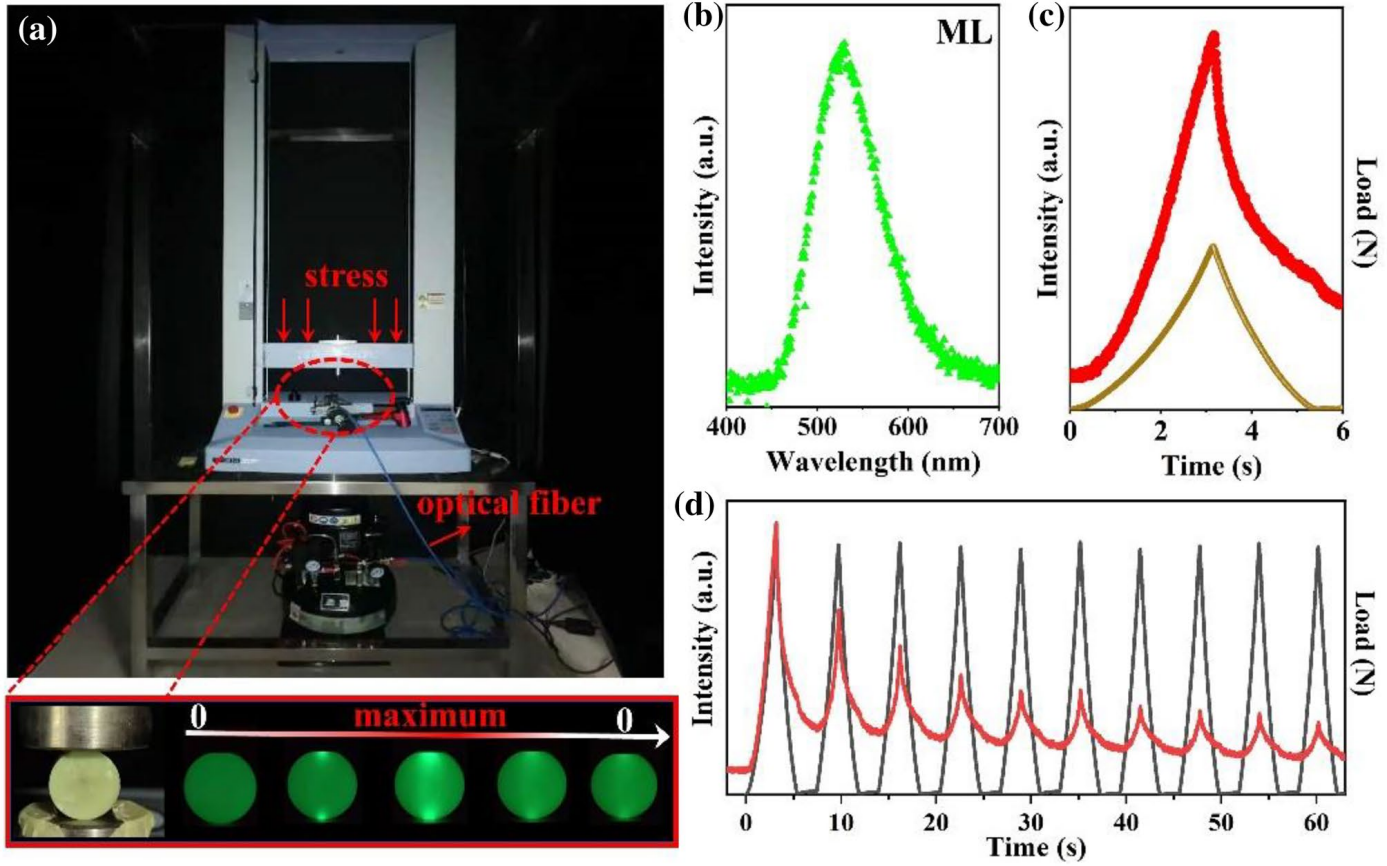
(**a**) Photo of lab-made system for the ML test and luminescent specimen as a function of stress. (**b**) ML spectrum of specimen. (**c**) ML curves when a load up to 1000 N was applied over time. (**d**) ML curves under the 1000 N compressive load for ten times.

Next, the ML property of specimen under external stress is examined. In order to analyze conveniently and accurately, a cylindrical specimen was made by the resin-based cementitious material and its ML luminescent properties were examined and are presented in Fig. 5. As shown in Fig. 5a, the system for the ML test is consisted of three parts: universal testing machine providing stress, optical fiber for collection and transmission of optical signal, as well as computer for control of testing machine and presentation of optical signal. The cylindrical specimen displays green emission without external stress due to the persistent luminescence. However, the emission intensity is significantly enhanced with the increase of stress at the middle part, and then reduces with the decrease of stress. The change of emission intensity indicates that the specimen equips with stress response.

Figure 5b exhibits the ML spectrum of specimen at room temperature. The observed ML emission spectrum shows resemblance to the PL and PRL spectra, which displays a wide asymmetric band, indicating that the ML emission is originated from the emission of phosphor. Figure 5c shows the ML intensity of cylindrical specimen during the load up to 1000 N. The ML intensity increases with the increase of compressive and reach the maximum at the same time, this phenomenon suggests that the relationship is linear between ML emission intensity and applied force, which is the basis for the non-contact detection of stress. Moreover, the ML repeated excitation of a mechanical pulse for 10 cycles under 1000 N was recorded and is presented in Fig. 5d. The ML intensity decreases gradually under the cyclic loading process, but the ML intensity still can be detected and the strongest point of ML intensity is simultaneous with the maximum load in each cycle. The trend of ML intensity is consistent with the change of load. These phenomena suggest that the cementitious material shows highly reproducible ML performance after molding and can be applied into visible stress monitoring and recording.

**Figure 6:**
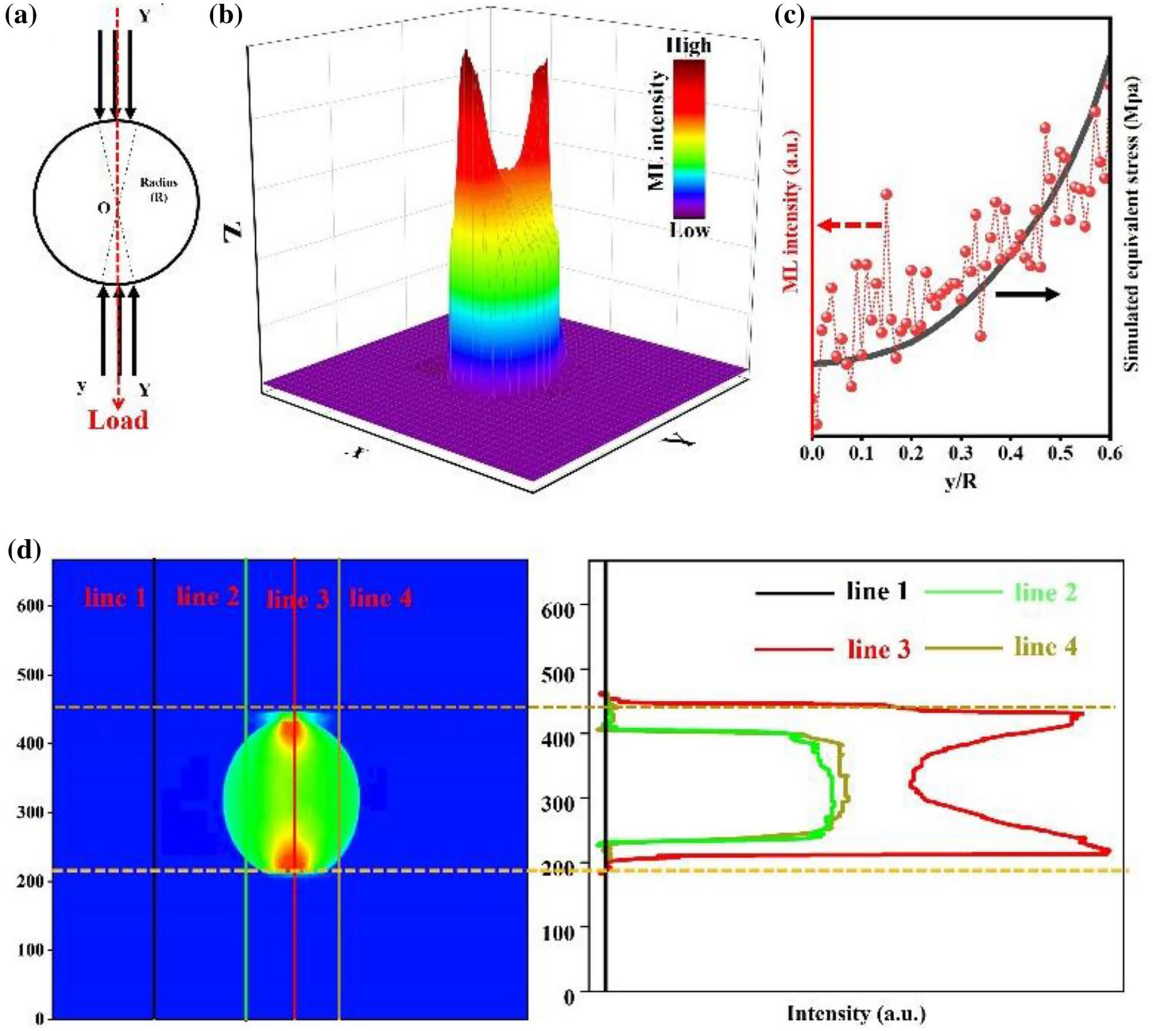
(**a**) The stress distributions in the cylindrical specimen. (**b**) The 3D ML distribution. (**c**) The comparisons between experimental and stimulated stress distribution along Y′OY in pellet under a compressive. (**d**) The change of ML intensity in different area.

The above results prove that the synthesized cementitious material can automatically emit visible light under external stress after molding, and the ML emission intensity displays linear relationship with the applied stress. In order to realize the precise detection of stress, the corresponding relationship between the stress distribution on the specimen and ML emission is analyzed in detail. The distribution of stress on the cylindrical specimen is calculated by finite element numerical simulation method. As shown in Fig. 6a, the stress distribution is along the direction of applied load (Y′OY direction), y is the distance from the center O along the Y′OY direction and R is the radius of the specimen, the simulated result indicates that the stress decreases gradually from the edge to the center. Besides, the ML intensity of the cylindrical specimen is collected and presented by the 3D model (Fig. 6b). It can be found that the emission intensity of central part is higher than that of the other parts. Moreover, the ML intensity of central part displays a trend of decreasing first and then increasing from one side of the cylindrical to the other side, the center point shows the lowest ML intensity, which is consistent with the distribution of stress on the specimen. The ML intensity (red dots) plotted versus y/R displays exponentially consistency with the simulated stress along the Y′OY direction (black line) as illustrated in Fig. 6c. In addition, the contour profile figure further shows the change of intensity in different areas. As shown in Fig. 6d, line 1 corresponds to the background area, where the emission intensity cannot be detected. Line 2 is the left area of the cylindrical specimen, where the emission intensity is steady, which is originated from the long afterglow emission. Line 3 crosses the central part, where the intensity decreases first and then increases. Line 4 is the right area of the cylindrical specimen, which shows a similar phenomenon to the line 2. The results are consistent with the brightness image as shown in Fig. 5a, which indicates that the ML emission can only be observed in the region under external stress, and the ML intensity is proportional to the magnitude of stress. These exciting results suggest that the value of stress can be estimated by measuring the ML intensity in the specimen, meanwhile, the ML emission can be observed immediately when the stress is applied, which demonstrates that the cementitious material realizes visible stress monitoring and recording.

**Figure 7:**
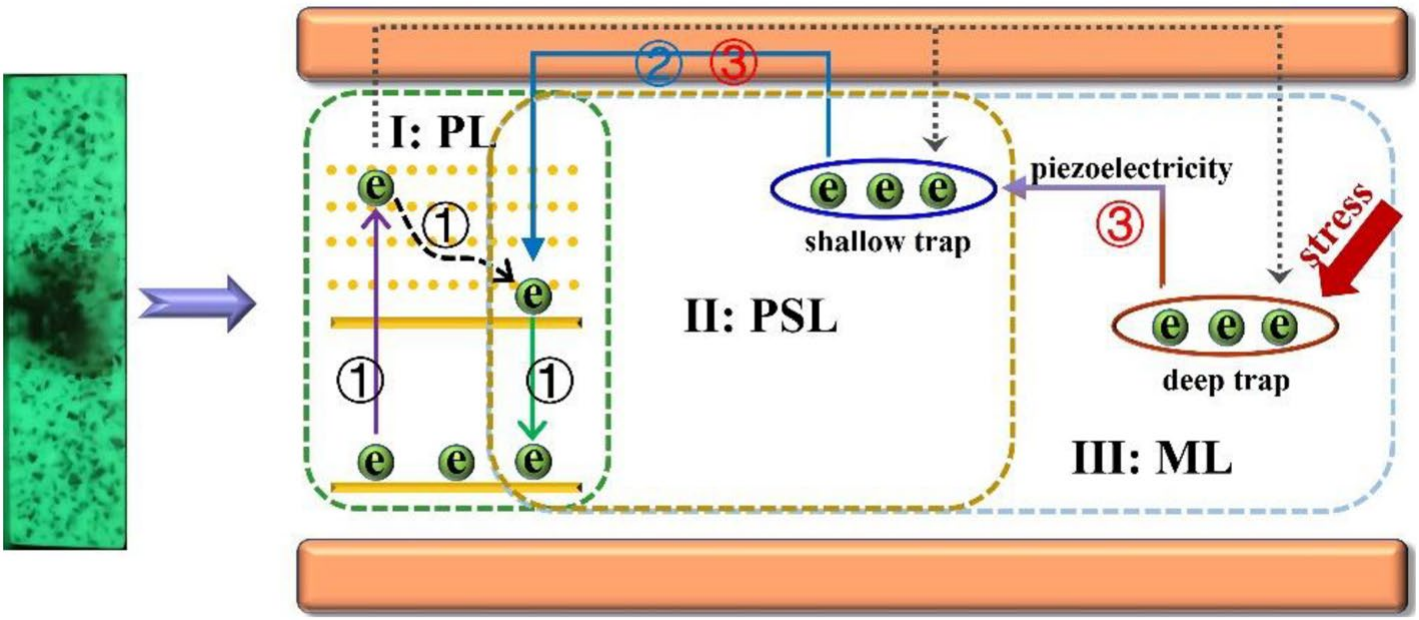
The luminescent mechanism of PL, PRL and ML.

The above results indicate that there are three kinds of luminescent model in the specimen: (I) PL; (II) PSL and (III) ML, the mechanism of each model is different^28,39,40^. As shown in Fig. 7, the PL process is assigned to the electrons transition between the excitation and ground orbital of Eu^2+^. With the help of UV excitation, the electrons in the 4f^7^ ground state of Eu^2+^ can transmit to the 4f^6^5d excited states, and then return to the ground state (process ①), accompanied with PL emission. However, some electrons can transmit to conduction band under the UV excitation, which will be captured by the traps when the electrons fall back from the high energy state (gray dashed). As for the electrons captured by the shallow trap, they can return to the luminescent center at room temperature under the effect of thermal disturbance, leading to PSL emission (process ②). It can be seen that the spectral shapes of PL and ML are similar, which prove that the emission of ML also come from Eu^2+^ ion. The structure of ML phosphor is asymmetric, the generated elastic deformation of specimen will cause the ML phosphor produce piezoelectric field when the stress is applied, which can excite the electrons from deep trap to the shallow trap and lead to ML emission (process ③)^40^. It can be seen that each luminescent mode corresponds to different mechanism, so that the specimen has multiple emission modes and realizes persistent emission and visual monitoring of stress.

**Figure 8:**
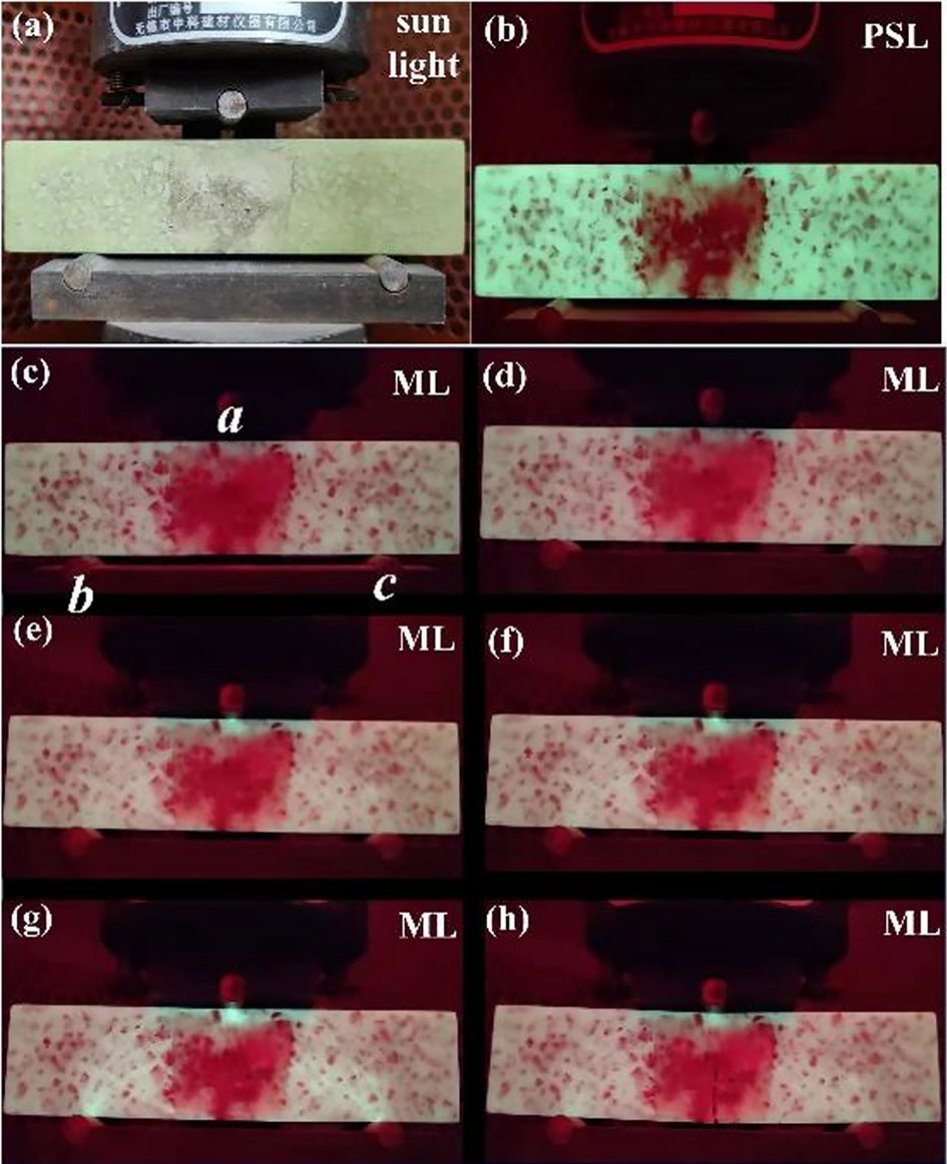
The PSL(**a**, **b**) and ML(**c**–**h**) photos of 4 cm × 4 cm × 16 cm specimen in bending test.

Based on the above researches, the ML phenomenon of the 4 cm × 4 cm × 16 cm specimen fabricated by the cementitious material was examined during the bending test. The luminescent images of specimen were collected and are presented in Fig. 8. The whole test was carried out in the presence of red interference light. The specimen displays green afterglow emission as shown in Fig. 8b, which can be observed in case of external interference. With the increase of pressure, *a* point emits visible green emission firstly as illustrated in Fig. 8d–f, indicating that the pressure on point *a* is bigger than that on points *b* and *c*. As the pressure continues to increase, the green emission can be found at points *b* and *c* (Fig. 8g). This phenomenon indicates that the pressure is different on the three points under bending test. Moreover, the splitting position of specimen corresponds to *a* point. It should be noted that the ML phenomenon can only be clearly observed in the three contact parts during the whole test process, and the ML result cannot be affected by the interference light. Besides, the change of luminescent intensity is consistent with the pressure, which proving that the external stress can be observed by visible light. Furthermore, the flexural and compressive strengths of the specimen after phosphor doping are 15.98 Mpa and 24.75 Mpa respectively, which are close to that of initial specimen, indicating that the doping of phosphor displays little impact on the strength.

## Conclusion

In summary, novel multifunctional cementitious material was synthesized via hierarchical aggregation smart ML phosphor and resin material. The flexural strength and compressive strengths of cementitious material could be enhanced by the doping of 0.5 vol‰ PP + 1.0 vol‰ steel + 1.0 vol‰ basalt hybrid fibers through suppressing the generation and development of micro and macro cracks. Moreover, the optimized resin-based cementitious material presented three kinds of luminescent modes via the addition of smart ML phosphor. The PL spectrum indicated that the specimen displayed green emission, and the decay curve showed that the green emission could last more than 40 min. Besides, the specimen possessed ML performance, which could emit obviously visible emission under stress. Finite element numerical simulation and profile analysis of contour map suggested that the visible emission could only be observed in the region affected by the external stress. Furthermore, the generation of visible light was synchronous with the action of external stress, and the change of emission intensity was proportional to the magnitude of stress. These results demonstrated that the synthesized cementitious material could be applied in the engineering fields such as bridges and tunnels, which could simultaneously achieve visual detection of strain distribution in stress concentrated areas and emergency lighting. This kind of function was realized for the first time in resin-based cementitious material, which was helpful for the innovation of stress examination technology and modern building materials.

## Methods

### Synthesis of resin-based cementitious materials

The raw materials for resin-based cementitious specimen were as follows, resin, curing agent, defoamer, rubber particle (0.2–0.4 mm) and fiber. The resin and curing agent were produced in China Korea (Wuhan) Petrochemical Co., Ltd. Fibers were bought from Beijing futen Technology Co., Ltd. The resin was 128 epoxy resin with transparent liquid, density: 1.16 (g/cm^3^, 25 °C), epoxy equivalent: 184–190 (g/EQ), viscosity: 12,000–15,000 (CPS, at 25 °C). The curing agent was modified amine TX–B2 with light yellow to brownish yellow transparent liquid, density: 0.97 ~ 1.03 (g/cm^3^, 25 °C), viscosity: < 300 (mPa.s, 25 °C), theoretical active hydrogen equivalent: 65–70 g/active H. The properties of fiber were shown in Table 1. The mix proportion of specimens were listed in Table 2.

**Table 1:** The properties of fiber.

	Length diameter ratio	Density (g/cm^3^, 25 °C)	Elongation (%)	Tensile strength (MPa)
polypropylene fiber (PP)	396	0.91	15–35	≥ 400
steel fiber	80	7.8	1.1–1.5	≥ 600
basalt fiber	100	2.85	3.1–3.2	≥ 800

The resin, curing agent and defoamer were firstly mixed, and then rubber particles and fibers were added successively. The mixed materials were poured into the standard mold (40 mm × 40 mm × 160 mm) for curing 7 days at room temperature, then examine the compression and flexural strengths.

**Table 2:** The mix proportion of specimens.

Resin (wt%)	Curing agent (wt%)	Defoamer (wt%)	Rubber particle (wt%)	PP (‰)	Steel (‰)	Basalt (‰)
100	30	3	400	0	0	0
100	30	3	400	0.5	0	0
100	30	3	400	1.0	0	0
100	30	3	400	1.5	0	0
100	30	3	400	2.0	0	0
100	30	3	400	0	0.5	0
100	30	3	400	0	1.0	0
100	30	3	400	0	1.5	0
100	30	3	400	0	2.0	0
100	30	3	400	0	0	0.5
100	30	3	400	0	0	1.0
100	30	3	400	0	0	1.5
100	30	3	400	0	0	2.0
100	30	3	400	0.5	0.5	0
100	30	3	400	0.5	1.0	0
100	30	3	400	0.5	1.5	0
100	30	3	400	0.5	2.0	0
100	30	3	400	0.5	0	0.5
100	30	3	400	0.5	0	1.0
100	30	3	400	0.5	0	1.5
100	30	3	400	0.5	0	2.0
100	30	3	400	0.5	1.0	0.5
100	30	3	400	0.5	1.0	1.0
100	30	3	400	0.5	1.0	1.5
100	30	3	400	0.5	1.0	2.0

### Preparation of mechanoluminescence specimen

To evaluate the mechanoluminescence property of specimen, the commercial strontium aluminate-type ML material (Youyan rare earth new materials Co., Ltd) was added into the resin-based cementitious materials, the mass ratio between ML material and resin-based cementitious material is 0.2:1. The cementitious specimen was prepared by using the standard mold (40 mm × 40 mm × 160 mm) in mechanical strength and self-made plastic mold (diameter, 25 mm; thickness, 15 mm) in luminescent test. The resin, curing agent and phosphor were mixed firstly via appropriate proportion, and then the mixture was poured into the bottom of the mold. After that, the resin-based cementitious material (synthesized in 2.1 section) was filled the remaining space of the mold. The cementitious specimen was cured at room temperature 7 days.

### Characterizations

The mechanical strengths of the specimens were examined by the pressure testing machine TYE-300 purchased from Wuxi Jianyi Instrument Machinery Co., Ltd. The test process followed the P.R.C standard ‘‘GB/T 17,671–1999^29^. The photoluminescence excitation (PLE), photoluminescence (PL) and persistent luminescence (PSL) spectra were detected by fluorescence spectrometer (FP-8600, JASCO Co., Japan), equipped with a 150 W Xe lamp. The mechanoluminescence (ML) intensity of specimen under extra stress was measured with a lab-made system comprising a universal testing machine (AGS-X10kN, Shimadzu Corp., Japan) and a photomultiplier tube (C13796, Hamamatsu Photonics, Japan). The ML spectrum of specimen was examined by fiber spectrometer (QE Pro, Ocean Optics) collocated with the universal testing machine.

## Data Availability

Experimental data for this study can be obtained from the Shiqi Liu on reasonable request.
